# Experiences of telehealth-delivered occupational performance coaching among Palestinian mothers of autistic children: a qualitative study

**DOI:** 10.3389/fped.2026.1974022

**Published:** 2026-10-07

**Authors:** Mohammad Hassan Salahat, Hassan Izzeddin Sarsak, Hatem Ahmad Al Adam

**Affiliations:** 1 Department of Occupational Therapy, Faculty of Allied Medical Sciences, Arab American University, Jenin, West Bank, Palestine; 2 Occupational Therapy Program, Batterjee Medical College, Jeddah, Saudi Arabia; 3 Bethlehem Directorate of Education, Ministry of Education, Al-Zeer Secondary School, Bethlehem, Palestine

**Keywords:** autism, occupational performance coaching, occupational therapy, Palestine, parent coaching, participation, qualitative research, telehealth

## Abstract

**Background:**

Families of autistic children in Palestine face substantial barriers to sustained, family-centred rehabilitation, including travel demands, fragmented services, financial pressures, digital inequalities, and recurrent movement restrictions. Occupational Performance Coaching (OPC) is a collaborative, occupation-focused approach that supports caregivers to analyse everyday participation challenges, identify priorities, generate contextually relevant strategies, and evaluate change. This study explored how Palestinian mothers experienced an eight-session OPC telecoaching intervention and how they perceived its acceptability, benefits, challenges, and contextual fit.

**Methods:**

A qualitative descriptive study was embedded within an explanatory sequential mixed-methods external pilot trial. All 12 mothers who completed OPC participated in a post-intervention semi-structured interview in Arabic via Zoom. The interview guide was informed by the Theoretical Framework of Acceptability (TFA) and included broader questions about intervention processes, perceived change, and contextual experience. Analysis of the complete Arabic interview dataset used reflexive thematic analysis. TFA provided deductive sensitizing concepts, while coding and theme development remained open to meanings arising from participants' accounts. Reflexive notes, cross-case comparison, attention to divergent accounts, peer debriefing, and an audit trail supported analytic reflexivity and transparency. COREQ guided reporting.

**Results:**

Five interrelated themes were developed: (1) from receiving advice to becoming a reflective problem-solver; (2) coaching in the context of everyday life; (3) taking responsibility: empowering, but demanding; (4) context-responsive access with conditional digital and cultural fit; and (5) meaningful progress as partial, variable, and still unfolding. Mothers valued collaborative reasoning, individualized goals, and the ability to work within real family routines. Remote delivery reduced some travel, scheduling, cost, and safety burdens, but connectivity, time, caregiving demands, and wider structural conditions remained important constraints. Increased parental responsibility could be experienced as both meaningful and demanding, and perceived changes were not uniform across goals or families.

**Conclusion:**

Mothers valued OPC telecoaching not only as a means of remote access, but as a way of working collaboratively on occupational challenges within everyday family life. Telehealth reduced selected access burdens in the Palestinian context but did not remove digital, caregiving, or structural constraints. The findings support further evaluation of culturally and contextually responsive, participation-focused telecoaching within pediatric occupational therapy.

## 1 Introduction

Supporting participation in everyday occupations is a central concern in pediatric occupational therapy. For autistic children, participation challenges may arise across self-care, play, social participation, family routines, and community mobility, often alongside communication and behavioural challenges that affect engagement in these occupations. Family-centred pediatric rehabilitation therefore increasingly emphasizes support that connects professional input with the routines and contexts in which participation occurs. Occupational Performance Coaching (OPC) was developed as a collaborative approach that supports caregivers to identify meaningful goals, analyse occupational performance, generate and test strategies, and evaluate progress rather than simply receive prescriptive advice (1, 2). Recent occupational therapy literature continues to position coaching as an important means of strengthening caregiver capacity and participation-focused practice (3).

This orientation is consistent with broader family-centred coaching in early intervention and pediatric rehabilitation. Within occupational therapy, OPC applies coaching to meaningful occupations and participation problems rather than treating coaching as a separate technique or a purely educational service. The practitioner works with the caregiver to identify priorities, examine occupational performance within person-task-environment conditions, reflect on possible explanations, and develop and adapt parent-generated actions. Coaching is distinguished from didactic parent education by its emphasis on partnership, reflection, shared decision-making, and the caregiver's expertise in the child and family context (4, 5). This distinction is important in autism intervention, where programs described as parent coaching vary considerably in the extent to which parents genuinely participate in planning, reflection, and decision-making (6). OPC is explicitly relationship- and occupation-centred: the practitioner does not simply transfer techniques but supports the caregiver to reason through problems and develop strategies that can be adapted across daily situations (1, 2).

Telehealth has created new opportunities to deliver coaching within children's natural environments. Telehealth refers to the mode of remote service delivery, whereas telecoaching refers to the collaborative intervention process conducted through that mode. Occupation-based telehealth coaching with families of young autistic children has shown promising changes in parent efficacy and child participation (7), while qualitative work has highlighted compatibility with everyday family life, collaborative parent-therapist relationships, and growing parent confidence (8). A randomized Iranian study of telehealth-delivered OPC reported improvements in occupational performance, goal attainment, and parent self-efficacy, although broader outcomes were mixed (9). More recent work in the United Kingdom suggests that online OPC can be acceptable and feasible for parents of children with disabilities, while also drawing attention to therapist preparation, fidelity, and implementation conditions (10). Considered together, these studies indicate that telehealth-delivered OPC is being examined across more than one national and clinical context, although the evidence remains limited and unevenly distributed. Systematic evidence on parent-mediated autism telehealth is encouraging but heterogeneous. Meta-analytic findings suggest benefits for parent implementation and self-efficacy, whereas effects on child outcomes vary across intervention models and studies (11, 12).

The context in which telecoaching is delivered matters. In the West Bank, Palestinian parents of autistic children have described financial strain, stigma, social isolation, limited autism-specific services, and substantial geographic and service-access barriers (13, 14). A Palestinian parent-focused intervention study indicates that structured family support is feasible and potentially beneficial in this setting (15), but family-centred occupational therapy coaching remains largely unexplored. Digital access is also uneven, and remote care must be interpreted alongside household connectivity and broader mobility constraints (16, 17). Thus, telehealth may reduce some barriers to care while creating or revealing others.

The present qualitative study formed part of a mixed-methods external pilot trial of an eight-session OPC telecoaching intervention for parents of autistic children in the West Bank. The quantitative trial findings are reported separately. This study aimed to explore Palestinian mothers' experiences and perceptions of OPC telecoaching following intervention completion, including its acceptability, perceived benefits and challenges, intervention processes, and the family, service, digital, and contextual factors that shaped participation and perceived change.

## 2 Methods

### 2.1 Study design, methodological orientation, and theoretical positioning

This qualitative descriptive study was embedded within an explanatory sequential mixed-methods external pilot randomized trial. The parent study adopted a pragmatic orientation, which supported using complementary methods to address practical questions about how a complex intervention could be delivered, experienced, and evaluated in a real-world service context (18). The parent trial evaluated the acceptability, feasibility, and preliminary outcome patterns of an eight-session OPC telecoaching intervention compared with a waitlist condition. The present article reports the post-intervention qualitative interviews conducted with mothers who completed OPC. Qualitative description was selected to provide a clinically useful, practice-relevant account that remained close to participants' everyday language and meanings while allowing interpretation of patterned experience across cases (19, 20).

The qualitative inquiry was informed by conceptually distinct methodological and theoretical elements. OPC and family-centred practice provided the intervention logic: collaborative goal identification, reflective occupational performance analysis, guided discovery, and iterative action were intended to strengthen caregivers' capacity to address participation problems within everyday routines (1, 2, 5). The Theoretical Framework of Acceptability (TFA) informed development of the interview guide and provided deductive sensitizing concepts during analysis. TFA conceptualizes acceptability through affective attitude, burden, ethicality, intervention coherence, opportunity costs, perceived effectiveness, and self-efficacy (21). Coding and theme development were not restricted to these constructs and remained open to meanings that did not map onto TFA categories. Ecological thinking supported interpretation of how family routines, service systems, digital infrastructure, community conditions, and broader sociopolitical circumstances shaped experience (22). These perspectives supported interpretation rather than functioning as fixed categories into which the data were forced.

Analysis followed a reflexive thematic approach (23, 24). The analysis remained grounded in participants' accounts but moved beyond simple topic summary to interpret patterned meanings across the complete interview dataset, while preserving variation and contradictory accounts. Coding agreement statistics were not used because these would be inconsistent with the reflexive analytic orientation (23, 24).

### 2.2 Parent study and OPC telecoaching intervention

The parent study was conducted with families of autistic children aged 3–7 years living in West Bank districts. The parent pilot trial was prospectively registered with ClinicalTrials.gov (NCT06427486). Fourteen parent-child dyads were allocated to OPC telecoaching and 12 to a waitlist condition; two parents allocated to OPC withdrew before the first session. The 12 parents who commenced OPC completed all eight sessions and constituted the qualitative sample for this paper.

OPC was delivered individually by videoconference over eight weekly sessions. Parents could initially identify more than three occupational performance problems and then prioritized up to three for formal COPM rating and use as coaching goals. Coaching began with the highest-priority goal, with other prioritized goals addressed as progress and family needs allowed. Across sessions, delivery followed the OPC domains of Connect, Structure, and Share, with the recurring process summarized in Table 1. Connect established a collaborative relationship through mindful listening, empathy, and partnership, while recognizing the parent's knowledge, strengths, and priorities. Structure moved from the selected occupational goal to collaborative analysis of current and preferred performance and the interaction among the child, the occupation, and the environment, followed by consideration of options, action planning, anticipated obstacles, and ways to evaluate or generalize the plan. Share involved brief, goal-linked information or resources when these supported the parent's reasoning and autonomy rather than replacing it. Parents then tried agreed actions within ordinary family routines between sessions, and the next session reviewed what had happened, what helped or remained difficult, and what the parent thought might explain the result. Plans could be retained, adapted, generalized to another situation, or replaced. Thus, occupations such as dressing, toileting, eating, communication, play, behavior, or safety entered the coaching process when they represented family-selected priorities rather than predetermined weekly topics. The Sanad Al-Hayaa Arabic digital platform supported registration, appointment booking, secure communication, and access to asynchronous materials. OPC was delivered as a parent-capacity-building intervention rather than as direct online treatment of the child. The child therefore did not need to remain on camera throughout sessions. The intervention followed established OPC principles and procedures (2).

**Table 1:** Common operational process across the eight-session OPC intervention.

Stage	Common OPC process	How occupational content entered	Between-session continuity
1. Goal prioritization	Parents identified occupational performance problems and prioritized up to three COPM goals; coaching began with the highest-priority goal.	The selected goal reflected a family-valued routine or occupation, such as dressing, toileting, eating, communication, play, behavior, or safety.	The prioritized goal became the focus for the next period of coaching and practice.
2. Connect	The coach used mindful listening, empathy, and partnership to understand the parent's perspective, recognize parent expertise and strengths, and establish a collaborative focus.	The parent described what mattered, the current situation or routine, and the desired change in participation or performance.	The agreed focus was carried into ordinary family routines between sessions.
3. Structure	Parent and coach established or clarified the valued goal, collaboratively analyzed current and preferred performance and person-task-environment factors, considered options, planned action, anticipated barriers, and identified ways to evaluate or generalize the plan.	Strategies were tailored to the child, occupation, family priorities, and the circumstances in which the occupation occurred.	Parents tried the agreed actions during daily routines and observed what helped, remained difficult, or needed adaptation.
4. Share	When useful, the coach offered brief, goal-linked information or resources after first eliciting the parent's perspective, supporting autonomy and parent-led problem solving.	Information or resources were selected to support the family's reasoning about the specific occupational challenge rather than to prescribe a fixed technique.	Information informed action and reflection while the parent remained the primary decision-maker.
5. Review and continue	At the next session, the parent and coach reviewed what was tried, what happened, and what the parent thought explained the result. Plans were retained, adapted, generalized, or replaced, and other prioritized goals were addressed as appropriate.	Occupational priorities could remain the same, broaden to another situation, or shift to another prioritized goal according to progress and family needs.	Review fed directly into the next coaching cycle, allowing iterative action and adaptation across the eight sessions.

OPC was individualized rather than delivered as a fixed eight-topic curriculum. The three enabling domains of Connect, Structure, and Share could recur within and across sessions; the table summarizes the common operational process across the eight sessions, and specific occupations were selected according to each family's prioritized goals.

Intervention fidelity was examined in the parent pilot trial using recorded-session sampling. Among sessions for which parents had consented to recording, three sessions from three different participants were randomly selected within prespecified early and late periods: two from the first intervention week and one from the final week. Two occupational therapy professors with expertise in early intervention and coaching independently reviewed each selected recording using the OPC Fidelity Measure and OPC CaseNote Audit Tool; neither assessor delivered the intervention. A prespecified criterion of ≥80% was used. All three sampled sessions met the criterion. OPC Fidelity Measure scores ranged from 88.9% to 100% for both assessors, while OPC CaseNote Audit Tool scores ranged from 84.6% to 92.3% for Assessor 1 and 85.0% to 92.3% for Assessor 2. No formal inter-rater reliability coefficient was calculated, so these results are not presented as evidence of inter-rater agreement. Rather, they provide targeted evidence of implementation at sampled early and late intervention points (25).

### 2.3 Participants and sampling

The qualitative sample comprised all 12 mothers who completed the eight OPC sessions. All 12 were invited to a post-intervention interview and all agreed to participate; there was no qualitative non-participation among intervention completers. During the final coaching session, parents were informed that an independent occupational therapist would contact them to invite them to discuss their experiences of the program. The interviewer subsequently contacted each mother, arranged a convenient interview time, and the mother booked the interview with her through the Sanad Al-Hayaa platform.

The qualitative component did not use a separate maximum-variation sampling strategy. All 12 intervention completers were invited and all agreed to participate because they had completed the full eight-session OPC intervention and could reflect on the entire program. Recruitment to the parent trial used two pathways. The Superhero Autism Foundation, an external community-based autism organization, shared study information with potentially eligible families in its network as a recruitment-outreach partner and was not involved in intervention delivery. Recruitment notices were also disseminated through social media and autism-related community pages, providing reach to families both connected with autism-related services and those outside formal referral pathways. The broader parent study therefore included families entering through different community and service-network routes, but the qualitative sample consisted of all 12 OPC completers rather than a separately constructed diversity sample. Information on whether participants had previously received services from, or had established professional relationships with, the intervention team or institution was not systematically collected in the parent trial; therefore, prior service use or relationships cannot be reliably characterized in this study. In one interview, the mother's husband was present and participated in parts of the discussion. He was not recruited or treated as an additional participant. Clearly attributable paternal contributions were retained in the analytic corpus, joint contributions remained jointly attributed, and ambiguous turns were not reassigned to the mother. No separate paternal case or theme was developed. Because all intervention completers were invited, data saturation was not used as a criterion to determine sample size or cessation of recruitment.

### 2.4 Research team and reflexivity

Interviews were conducted by Amira Awda, a female occupational therapist with a BSc in Occupational Therapy and substantial experience interviewing parents through her university and professional practice. Before formal data collection, she received study-specific training from the principal investigator on the qualitative interview protocol and practised the interview with a volunteer parent who was not included in the qualitative sample. As part of the parent trial, she had previous study-related contact with participants through administration of the Canadian Occupational Performance Measure, but she was not involved in delivering the OPC intervention. Her separation from intervention delivery reduced the likelihood that mothers were directly evaluating the program to the therapist who had provided their coaching, although her prior assessment contact with participants was considered as part of the interview context.

The principal investigator had delivered the OPC intervention and was therefore familiar with participants' goals and coaching experiences. This provided contextual knowledge but also created the possibility of positive expectancy and interpretive bias. Hatem Ahmad Al Adam, a social worker and member of the research team, contributed to transcription and maintained reflexive notes jointly with the principal investigator during transcription and analysis. These notes documented developing assumptions, interpretive questions, and alternative readings and were revisited against the full transcripts, particularly when participant accounts challenged initially positive or uniform interpretations. The notes informed reflexive scrutiny and analytic dialogue but were not treated as an independent source of participant data.

### 2.5 Interview guide and data collection

A semi-structured interview guide was developed in Arabic. Questions explored enrolment and participation, experiences of the Sanad Al-Hayaa platform and Zoom, the coaching relationship and session process, perceived changes for parent and child, fit with family routines, privacy and cultural acceptability, financial and transportation implications, political and safety constraints, burden, preferred dose, recommendations, and willingness to continue or recommend the service. The guide was informed by TFA constructs while allowing participants to raise experiences outside the framework (21). Before formal data collection, the guide and interview procedures were piloted by the interviewer with one volunteer parent who was not included in the qualitative sample, allowing the interviewer to practise the protocol and minor refinements to be made for clarity. The complete English translation of the interview guide is provided in Supplementary Material 1.

Interviews were conducted in Arabic via Zoom from 15 June to 7 July 2025, after completion of the intervention, and lasted approximately 35–60 min. Interviews were audio-recorded with permission. No repeat interviews were conducted; one interview was rescheduled. Participants were offered the opportunity to review their transcript. Most declined because they were comfortable with what they had reported; one mother reviewed her transcript and confirmed its accuracy. Participants were not asked to validate the final themes.

### 2.6 Data analysis

Recordings were transcribed verbatim in Arabic and checked against the audio. Analysis was conducted on the complete Arabic interview dataset. The interview guide was used as a map for understanding the context in which accounts were elicited, but interview domains were not treated as predetermined themes. Participant speech was distinguished from interviewer questions, summaries, examples, and prompts. Prompting context was considered during interpretation, particularly where responses consisted primarily of agreement with a specific interviewer proposition.

The analysis proceeded through repeated familiarization, coding of meaningful participant segments, within-case summaries, cross-case comparison, development of candidate patterns, review of thematic boundaries and contradictory cases, and refinement of final themes, broadly following Braun and Clarke's reflexive thematic analysis approach (23, 24). Initial coding combined semantic attention to what mothers explicitly described with interpretive attention to processes such as changing parental reasoning, ownership, contextual fit, effort, and perceived change. TFA concepts were considered as deductive sensitizing ideas, but codes and themes were not required to map onto TFA categories. Coding, memo-writing, categorization, and organization of coded segments were supported using Taguette, an open-source qualitative data-analysis platform (26).

Hatem Ahmad Al Adam independently coded two transcripts and discussed coding and interpretation with the principal investigator. This was used for reflexive dialogue, consideration of alternative interpretations, and conceptual clarification rather than calculation of inter-rater reliability or forced coding consensus. He also contributed to reviewing emerging codes, the final themes, and selected quotations. The remaining transcripts were coded by the principal investigator. Peer debriefing with Hassan Izzeddin Sarsak, a senior occupational therapy researcher and doctoral supervisor, supported critical examination of developing themes, including whether they were coherent, analytically distinct, and adequately supported by the dataset. An audit trail documented coding decisions, theme development, cross-case comparisons, negative and divergent cases, and quotation selection. Theme development proceeded iteratively through within-case and cross-case comparison; candidate themes were repeatedly reviewed against the complete dataset and refined so that each represented a coherent patterned meaning rather than an interview-guide topic. Five final themes were developed.

### 2.7 Cross-language analysis and translation process

The interviews were transcribed, coded, and interpreted in Arabic; the full dataset was not translated into English for analysis. Codes and candidate themes were developed from the Arabic material, with English theme labels formulated during manuscript preparation to represent the underlying analytic concepts. Quotations selected for publication were forward-translated into English by the principal investigator and reviewed by Abdelrahman Awad, a bilingual non-study contributor, for linguistic accuracy and cultural equivalence. Translation aimed to preserve meaning, tone, and pragmatic intent rather than impose literal word-for-word equivalence. Participant codes are used to protect anonymity.

### 2.8 Trustworthiness and reporting

Trustworthiness was supported through an explicit audit trail, repeated engagement with the complete dataset, attention to interviewer influence and prompting context, within-case and cross-case comparison, independent coding and reflexive discussion of two transcripts, peer debriefing, and deliberate examination of negative and divergent cases. Reflexive notes maintained jointly by the principal investigator and Hatem Ahmad Al Adam were revisited to identify where early interpretations converged with, overstated, or were contradicted by participants' accounts. The analysis did not seek statistical representativeness or treat frequency as the sole indicator of analytic importance. COREQ guided reporting of the study and a completed checklist is provided as Supplementary Material 2 (27).

### 2.9 Ethical considerations

Ethical approval for the parent study was granted by the Arab American University Institutional Review Board (R-2024/A/164/N). All parents provided written informed consent before enrolment and reaffirmed consent for the qualitative interview and recording. Participation was voluntary, and parents were informed that their responses would not affect access to services or their relationship with the study team. Participant identifiers were replaced with study codes before analysis, and electronic data were stored on password-protected systems accessible only to authorized members of the research team.

## 3 Results

All 12 mothers who completed OPC took part in an interview. The mean maternal age was 34.0 years (SD = 5.0). Six families lived in cities, five in villages, and one in a refugee camp. The mean child age was 5.50 years (SD = 1.16). Parent-reported autism severity in the qualitative demographic questionnaire was mild for six children, moderate for five, and severe for one; these categories were distinct from the independently assessed CARS-2 classifications used in the quantitative pilot trial. One husband was present with his wife during one interview. Five interrelated themes were developed across the complete interview dataset, describing how mothers experienced the coaching process, its relationship to everyday family life, the responsibility it placed on them, the fit of remote delivery within the Palestinian context, and the nature and limits of perceived change. Table 2 summarizes selected maternal and child characteristics, and Table 3 presents the final thematic structure with illustrative quotations.

**Table 2:** Selected characteristics of mothers and children in the qualitative sample (*n* = 12).

Characteristic	Qualitative sample (*n* = 12)
Maternal age, years, mean (SD)	34.0 (5.0)
Residence, *n* (%)	
City	6 (50.0)
Village	5 (41.7)
Refugee camp	1 (8.3)
Child age, years, mean (SD)	5.50 (1.16)
Parent-reported autism severity, *n* (%)	
Mild	6 (50.0)
Moderate	5 (41.7)
Severe	1 (8.3)

Values are presented as mean (SD) or *n* (%). Autism severity categories are parent-reported, based on mothers’ responses to the demographic questionnaire, and are distinct from the independently assessed CARS-2 classifications reported in the quantitative pilot trial.

**Table 3:** Qualitative themes, subthemes, and illustrative quotations.

Theme	Subthemes	Illustrative quotation
1. From receiving advice to becoming a reflective problem-solver	Prioritizing; analysing barriers, facilitators, and strengths; parent-generated solutions; ownership and capability	“When I took him to a center … they gave sessions and told me to do certain things … but here I started thinking about what I can do.” (P4)
2. Coaching in the context of everyday life	Everyday occupations and family priorities; home-context fit; practice and generalization; complementarity with centre-based care	“A lot of times, the child is acting differently at the center than at home. Telecoaching brings the perspective of the therapist closer to the real problems we face at home.” (P10)
3. Taking responsibility: empowering, but demanding	Ownership and accountability; meaningful responsibility; time, fatigue, and competing roles; sustained effort	“There is more responsibility on me as a mother, but it's also empowering.” (P8)
4. Context-responsive access with conditional digital and cultural fit	Flexibility, travel, and cost; safety and mobility; privacy and cultural fit; digital friction and conditional access	“The program is excellent and very suitable for families in the current conditions in the West Bank … It saves time and effort for parents.” (P9)
5. Meaningful progress as partial, variable, and still unfolding	Functional gains; goal-specific change; gradual or non-linear progress; unresolved barriers; variable family effects	“Not all goals were fully achieved, but we moved forward step by step.” (P1)

Quotations were translated from Arabic. Participant identifiers are study codes. Ellipses indicate omitted nonessential wording without changing the intended meaning.

### 3.1 From receiving advice to becoming a reflective problem-solver

The strongest cross-case pattern concerned a change in how mothers approached everyday problems. Mothers did not describe OPC simply as receiving information about autism or collecting techniques from a therapist. Across accounts, they described learning to identify what mattered most, examine why a routine was difficult, notice barriers as well as strengths and resources, and consider what they themselves could try. For some, this represented a clear movement away from a professionally directed model of care; for others, who already regarded themselves as capable and involved caregivers, coaching appeared to organize and deepen existing ways of thinking rather than create confidence from the beginning.

This shift was most visible when mothers contrasted OPC with previous experiences of being given professional instructions. One mother explained, “When I took him to a center … they gave sessions and told me to do certain things … but here I started thinking about what I can do” (P4). The same participant valued the analytical process itself: “I liked how it lets you think about barriers and facilitators, and find solutions, not just superficially teaching, but analyzing things deeply” (P4). Another mother described a similar change in perspective: “Honestly, I started to think of solutions from a different perspective for various problems or behaviors” (P5).

Goal prioritization and collaborative decision-making were part of this developing agency. Several mothers recalled initially wanting many things to change at once and described learning to identify a manageable priority and work through it step by step. Others emphasized being active contributors rather than passive recipients. As one mother put it, “I felt like an active partner in the meetings; we set goals and plans together” (P2). Attention to facilitators also encouraged some mothers to notice child strengths, environmental resources, and opportunities within the home rather than focusing only on difficulty.

The pattern was not a uniform transition from low to high confidence. One mother explicitly described herself as confident before the program and resisted the idea that OPC had changed her personality or general confidence; what changed for her was a better understanding of why her child behaved in particular ways. This divergent account helped define the theme more precisely. What was shared across cases was not parental inadequacy followed by empowerment, but a more structured capacity to analyse, choose, and act on everyday occupational problems.

### 3.2 Coaching in the context of everyday life

Mothers repeatedly connected the value of OPC to the fact that goals and strategies were developed around real occupations and routines. The home was not experienced merely as the place from which a video call occurred; it became part of the reasoning process. Difficulties with dressing, toileting, eating, communication, play, behaviour, and safety could be considered in relation to the people, materials, timing, and environmental conditions already present in family life.

This was especially clear when mothers compared what professionals could see in a centre with what parents encountered at home. One participant explained, “A lot of times, the child is acting differently at the center than at home. Telecoaching brings the perspective of the therapist closer to the real problems we face at home” (P10). Another mother described how small strategies could be inserted into ordinary activity rather than reserved for a therapy period: “It shed light on simple things … giving instructions. I can give instructions even while I'm in the kitchen. Sometimes there are simple things that just don't come to mind for a mother” (P7).

The individualized nature of planning was equally important. Mothers described contributing information about what was realistic for their child, home, and family circumstances rather than receiving a standardized program. Practising goals within daily routines gave some mothers repeated opportunities to support participation and generalization. One mother summarized this occupational fit directly: “Everything we worked on came from his daily life and our routines. It was not like a center where the child only sits and plays with blocks” (P11). The opportunity to practise, however, still depended on the mother's time, the child's availability, and whether the surrounding environment allowed the activity to be carried out safely.

Centre-based care was not rejected uniformly. Some mothers continued to value specialist therapists and described their children as responding well in centre settings. The distinction in their accounts was therefore not that telecoaching replaced professional therapy, but that OPC gave parents a different role and brought professional reasoning closer to problems occurring between appointments. In this sense, telecoaching was experienced as complementary to centre-based care rather than simply an alternative location for the same service.

### 3.3 Taking responsibility: empowering, but demanding

Parent-led coaching moved a substantial part of implementation into daily family life. Mothers were expected to try agreed plans, observe what happened, adapt their approach, and return to the next session ready to review progress. For some, this weekly rhythm created constructive accountability. One mother explained, “The fact that I had to present results weekly … motivated me to work with my son … and to push myself to work with him more” (P1). The same process, however, also made the extent of maternal responsibility more visible.

Several mothers experienced this increased role positively because it gave them greater ownership of change and allowed them to act throughout the day rather than wait for a clinic session. One participant expressed the tension directly: “There is more responsibility on me as a mother, but it's also empowering” (P8). Another, contrasting her earlier role with OPC, said, “I used to just follow, but now the effort is more on me” (P1). Increased responsibility was therefore not experienced only as burden. For some mothers it was closely tied to feeling more involved, capable, and able to influence what happened in everyday routines.

At the same time, parent-led action required time, energy, and practical opportunity. Employment, household responsibilities, kindergarten, other therapy appointments, care for family members, and fatigue could reduce consistency between sessions. One mother explained, “I wasn't consistent 100% because of these barriers, kindergarten and therapy sessions took up most of the day” (P12). Another summarized the same limitation more generally: “Plans were realistic but require time and energy. I was able to implement simple tasks” (P3). These accounts show that finding a strategy acceptable in principle did not guarantee that a family had sufficient capacity to enact it consistently.

Responsibility and agency therefore remained intertwined rather than mutually exclusive. The same parent-led structure could feel motivating in one circumstance and demanding in another, or both at once. Mothers' accounts suggested that ownership was most sustainable when goals were limited and meaningful, strategies fitted existing routines, expectations were realistic, and progress was visible enough to justify the effort being invested.

### 3.4 Context-responsive access with conditional digital and cultural fit

Remote delivery reduced practical demands that accompanied repeated access to rehabilitation. Mothers described avoiding transportation, choosing session times around household or work responsibilities, and receiving support without leaving home. Cost was explicitly important for some. One mother explained, “If I wanted to take my child to a doctor or a center for these things, it would definitely cost me. I would have to pay for sessions and transportation. Telecoaching definitely saved me these expenses” (P9). The same participant described the program as particularly suitable “in the current conditions in the West Bank,” adding that it saved parents time and effort (P9).

For some families, the political and security environment made remote access more than a question of convenience. One mother described a situation in which travel was disrupted by nearby military activity: “My husband called and told me not to go out because there was an army at the building. Situations like this happen a lot. When I can't make it to the center, I feel relieved there's another way I can make it up to my daughter through telecoaching” (P11). Other mothers similarly connected remote participation with reduced exposure to uncertain movement conditions. Telecoaching did not remove those structural restrictions, but it reduced dependence on travel for this component of care.

Privacy and cultural comfort were also part of how mothers judged the model. Most accounts were accepting, but comfort was not always immediate. One mother said, “At first it was a bit uncomfortable, but after a few sessions, I got used to it and it became normal. I think telecoaching respected my privacy and my family's culture. I didn't feel anything bad” (P11). Another emphasized the practical comfort of being at home: “For me, as a mother, I actually prefer remote services because you feel like you have more options. It's easier and more comfortable. You can take the session from home, your child is with you, and it fits your life better” (P7).

Digital participation was nevertheless conditional. Families who completed the intervention generally managed appointment booking and Zoom after familiarization, but some mothers reported intermittent internet problems or difficulty opening materials in the resource library. Preferences for asynchronous material also differed, including requests for more video-based resources. These accounts temper any conclusion that the digital model was universally easy: all participants in this study had already met the digital requirements for videoconferencing, so the experiences of families with more severe connectivity or device barriers were not represented.

### 3.5 Meaningful progress as partial, variable, and still unfolding

Mothers described meaningful changes during the intervention, particularly in parent-selected activities that were practised repeatedly in daily life. Their examples were concrete rather than global. One mother described toileting progress: “The program helped me with bathroom training. We had almost lost hope. Thank God, she has improved a lot … She is about 70% there now, which is a big achievement” (P12). Another described dressing: “We started with a goal for him to take off his clothes by himself. Before, he only moved his hand or foot. Now he can almost take them off on his own, and I only need simple help” (P11). A third reported that a child who had not previously answered his name did so “in more than 70% of attempts” after targeted practice (P10).

These perceived gains were neither uniform nor complete. Mothers distinguished goals that changed relatively quickly from those requiring prolonged repetition, and they described weeks in which progress stalled or appeared to move backwards. One participant summarized this temporal pattern simply: “Not all goals were fully achieved, but we moved forward step by step” (P1). Another described progress as slow but still meaningful: “It is nice to see progress. It is slow, but you live the moment and feel you achieved something” (P11). Some mothers felt that certain goals would require two or three months, or longer, rather than the eight-week intervention period.

The surrounding environment also placed boundaries on what could change. Safety-related goals were difficult to practise when streets were busy or neighbourhood conditions were perceived as unsafe. One mother stated, “I couldn't always take him outside to practice because our street is busy and the neighborhood is crowded” (P7). Another reported that she wanted to practise outdoors but could not always do so because of safety and traffic (P9). Broader parent and family effects also varied. Some mothers described calmer routines, greater hope, or reduced stress, whereas others reported no meaningful change in family life. The pattern was therefore one of proximal, goal-specific change rather than uniform improvement across all aspects of child and family functioning.

These accounts are reported as mothers' perceptions of change, not as independent evidence of intervention efficacy. Their value is explanatory: they show which changes parents noticed and valued, how those changes related to targeted occupations and repeated practice, and why progress could remain partial when time, opportunity, environmental safety, or family capacity constrained implementation.

Figure 1 presents an interpretive synthesis of the five themes and their relationships. Telehealth functioned primarily as an access pathway, whereas the OPC relationship supported an iterative process of reflection, prioritization and planning, action within everyday occupations, and review and adaptation. Mothers' experiences of this process were shaped by digital access, service availability, sociopolitical and mobility conditions, cultural fit and privacy, time, and family capacity. Greater parental responsibility could support ownership and capability while also increasing workload. Perceived change was therefore experienced as meaningful but goal-specific, variable, and still unfolding.

**Figure 1:**
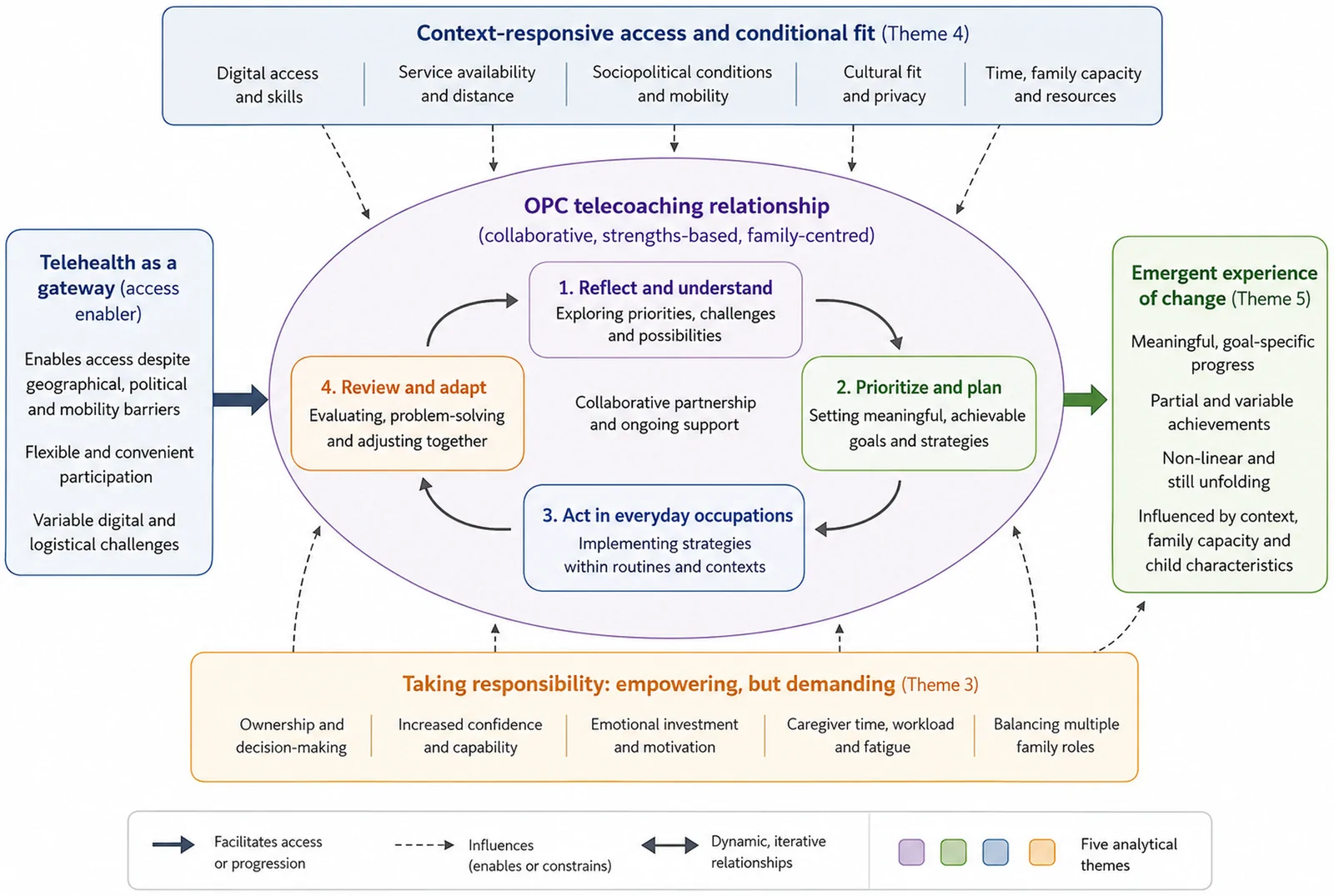
Interpretive thematic model of mothers’ experiences of occupational performance coaching telecoaching. The model synthesizes relationships among the five qualitative themes. Telehealth is represented as an access pathway to the collaborative OPC process, which involved iterative reflection, prioritization and planning, action within everyday occupations, and review and adaptation. Contextual conditions could enable or constrain engagement, while increased parental responsibility was experienced as both empowering and demanding. Perceived change was meaningful but variable and context-dependent. Arrows indicate interpretive relationships among themes and do not represent a tested causal pathway.

## 4 Discussion

### 4.1 Principal findings

This qualitative study explored how 12 Palestinian mothers experienced OPC delivered through telehealth after completing an eight-session intervention. Across the five themes, the central story was not simply that remote care was convenient or acceptable. Mothers described learning to analyse everyday participation problems more deliberately, embedding strategies within ordinary routines, and taking greater ownership of action between sessions. At the same time, this parent-led model generated additional work, and both access and perceived change remained conditioned by digital, family, environmental, and sociopolitical circumstances. The contribution of the study therefore lies in showing how the coaching process, the telehealth delivery mode, and the surrounding context interacted in mothers' accounts.

Two distinctions are especially important. First, the therapeutic value of working on occupations in the settings where they occurred was different from the practical value of avoiding travel to a centre. Second, greater parent agency was not synonymous with reduced burden. Mothers could experience more ownership and more work at the same time. These distinctions help move interpretation beyond a simple conclusion that telecoaching was acceptable and toward a more mechanism- and context-focused account of why it was valued, where it was difficult, and what kinds of change mothers noticed. Figure 1 summarizes this interpretive relationship among telehealth access, the OPC process, parent-led action in everyday occupations, and the contextual conditions that shaped enactment and perceived change.

### 4.2 Reflective parent agency as a mechanism of coaching

The shift from receiving instructions toward analysing, prioritizing, and generating solutions is closely aligned with the conceptual basis of OPC (1, 2). Earlier qualitative work with mothers receiving OPC described increased insight, strategy use, and engagement in problem-solving (28, 29), while occupational therapy telehealth research has similarly identified collaboration, everyday-life compatibility, and increased parental confidence or empowerment as valued features (7, 8). The recent UK study of online OPC also found that parents valued partnership and collaborative problem-solving, although therapist fidelity varied (10). Considered alongside the Iranian telehealth OPC study and other occupational therapy telehealth work cited above, our findings do not suggest that reflective parent agency is unique to Palestine. Rather, they extend an emerging international evidence base by showing how a recognisable OPC process could be enacted within an Arabic-speaking service context marked by different cultural, resource, digital, and mobility conditions.

The findings also add nuance to the language of empowerment. Not every mother entered the program feeling uncertain or dependent on professionals. Some already described themselves as confident and highly involved caregivers, and for them coaching appeared to organize, deepen, or redirect existing capability rather than create it. This is consistent with contemporary occupational therapy literature that treats parent-therapist collaboration as a family-centred practice involving shared planning, parent expertise, and participation in decision-making rather than professional transfer of knowledge alone (5, 30). The iterative sequence mothers described, identifying a real problem, trying an action, reflecting on what happened, and planning the next step, is also consistent with adult-learning principles described in coaching approaches (5), while repeated mastery and feedback provide a plausible route to perceived self-efficacy, a construct also represented in TFA (21). These theoretical parallels help interpret the findings but were not imposed as analytic frameworks. We therefore interpret Theme 1 as development of reflective agency and transferable problem-solving, not as a universal movement from low to high confidence. This distinction matters because family-centred coaching risks becoming paternalistic if parents are implicitly positioned as deficient before professional input.

### 4.3 Everyday occupation and contextualized intervention

The second theme highlights an occupational therapy contribution that is distinct from general telehealth convenience. Mothers valued being able to analyse dressing, toileting, communication-related participation, play, behaviour, and safety in relation to their own routines and environments. Little et al. found that occupation-based telehealth coaching could support parent efficacy and participation in parent-selected routines (7), and Wallisch et al. identified compatibility with everyday life as central to parents’ experience of occupational therapy telehealth (8). OPC research has likewise shown that caregiver-generated strategies are strongly shaped by environmental and contextual understanding (29). More recent reviews of family-centred occupational therapy consultation similarly indicate that caregiver understanding, confidence, and implementation are commonly strengthened when intervention is linked to natural environments, while noting the limited evidence from telehealth and non-Western settings (31).

This context-sensitive reasoning may explain why mothers sometimes contrasted OPC favourably with centre-based services without dismissing professional centre expertise. The issue was not simply whether the session occurred at home or in a clinic. Mothers described a perspective gap between what professionals might observe during a bounded centre encounter and what families encounter across daily routines. Telecoaching allowed professional reasoning to be informed by the parent's knowledge of those routines and by problems that might not be directly observable in a centre. Earlier rural autism research similarly suggested that remote services can augment rather than necessarily replace face-to-face supports, while reducing travel and increasing family capacity (32). Our findings therefore support a complementary model in which direct services and parent coaching perform different but potentially reinforcing functions.

### 4.4 Responsibility, caregiver capacity, and the limits of parent-led care

A central tension in the data was that the parent-led nature of OPC was both a source of value and a source of work. Mothers could experience greater responsibility as meaningful because they had more influence over goals, could act throughout the day, and could see the relationship between their own actions and change. Yet implementation competed with employment, other therapies, household responsibilities, fatigue, and care for other family members. The UK online OPC feasibility study likewise identified the practical importance of family capacity and between-session enactment (10). Broader telehealth parent-intervention evidence suggests that parent-mediated approaches can improve implementation fidelity, self-efficacy, or parenting practices, while effects on distress and broader family outcomes are more variable (11, 33, 34). Our findings add an experiential explanation for that variability: an intervention can increase capability while simultaneously increasing the amount of work expected from the caregiver.

This tension is especially salient because all interview participants were mothers. Palestinian qualitative research has documented substantial psychological, financial, and caregiving pressures among parents of autistic children in the West Bank (13), but the present study was not designed to compare maternal and paternal roles and should not be used to infer that mothers necessarily carry more responsibility than fathers. What it does show is that, for these mothers, family-centred practice cannot assume unlimited capacity simply because parents value involvement. Coaching therefore needs to negotiate dose, pacing, competing commitments, and what is realistically achievable between sessions. Supporting agency and protecting caregiver capacity should be treated as complementary goals rather than competing priorities.

### 4.5 Telehealth as service fit within the Palestinian context

Mothers' accounts of flexibility, reduced travel, cost savings, privacy, and safety are consistent with international telehealth and telerehabilitation literature (8, 32, 34–36), but the meaning of these advantages was shaped by the West Bank context. Earlier Palestinian research documented geographic, financial, and organisational barriers to autism-related services (14). During the period surrounding the study, the United Nations Office for the Coordination of Humanitarian Affairs documented a marked increase in physical movement obstacles across the West Bank, including checkpoints, road gates, earthmounds, and other closures that disrupted access between towns, villages, major roads, workplaces, and services (17). Against this background, remote coaching reduced dependence on travel for one component of rehabilitation. It did not remove the wider restrictions themselves.

Digital access created a second boundary. Palestinian Central Bureau of Statistics data indicate high household internet availability in Palestine, but household-level access does not guarantee stable connectivity, adequate devices, digital confidence, privacy, or equal ability to participate in videoconferencing (16). A scoping review of digital health in fragile and conflict-affected settings in the Middle East and North Africa similarly identified weak infrastructure, computer literacy, privacy, cultural constraints, and resource limitations as implementation concerns, while also finding that digital health can extend access and support consultation (37). This matters for interpretation of our positive usability accounts because the sample consisted of families who were able to enrol in and complete an online intervention. Future implementation should therefore include low-bandwidth options, technically simple booking and communication, accessible asynchronous materials, and alternatives for families who cannot reliably use video-based care.

### 4.6 Meaningful but bounded change

Mothers described the clearest changes in occupations and routines that were directly selected, practised, and reviewed. This pattern is consistent with occupation-based telehealth coaching and tele-OPC studies in which parent-selected performance goals have shown encouraging improvement (7, 9, 38). It is also consistent with the logic of OPC, where the most proximal change is expected in caregiver reasoning, strategy use, and opportunities for participation rather than uniform change across broad measures of autism characteristics or family quality of life (2). The recent scoping review of family-centred occupational therapy consultation similarly found the most consistent qualitative gains in caregiver understanding, confidence, and ability to implement strategies in natural environments (31).

The qualitative findings nevertheless argue against a simple success narrative. Some goals remained incomplete, some changed slowly or non-linearly, some could not be practised as intended, and broader family-level effects were inconsistent. Systematic reviews of parent-mediated telehealth show generally promising parent and child outcomes but substantial heterogeneity across interventions, outcomes, and study designs (11, 33, 34). A 2025 systematic review of telehealth early intervention also noted technological-literacy challenges and limited evidence across broader family outcomes (34). We therefore treat mothers' accounts as explanatory evidence about perceived change, not as proof of efficacy. Their value lies in showing why change may emerge first in specific occupations, why generalisation may depend on opportunities outside the home, and why an eight-week intervention may initiate new problem-solving processes without resolving every participation challenge.

### 4.7 Implications for pediatric occupational therapy

The findings suggest four practical implications for pediatric occupational therapy. First, telehealth should be distinguished from the intervention delivered through it: videoconferencing created access, whereas the OPC process involved collaborative goal-setting, reflective analysis, and parent-led action. Second, intervention evaluation should include participation and caregiver capability alongside symptom- or impairment-focused outcomes, because the changes mothers valued were often specific to daily occupations and routines. Third, parent-led intervention should be calibrated to caregiver capacity. Flexible appointment times do not make an intervention family-centred if between-session expectations are unrealistic. Fourth, culturally and contextually responsive telecoaching requires attention to language, privacy, provider relationships, digital accessibility, and the service and mobility conditions surrounding the family.

For Palestinian pediatric rehabilitation, OPC telecoaching may be most useful as a complement to existing centre-based or direct services rather than as a replacement. It can extend occupational therapy reasoning into daily routines and may preserve contact when travel becomes difficult, but it also depends on trained coaches, adequate technology, and realistic expectations of parents. Implementation research should therefore examine models that combine direct and coaching approaches, include families with weaker digital access, and test flexible or low-bandwidth delivery. Longer follow-up is also needed to examine whether parents continue to use reflective problem-solving with new occupational challenges after formal coaching ends.

### 4.8 Strengths and limitations

Several features strengthen the credibility and transparency of this study. All 12 mothers who completed OPC participated in the post-intervention interviews, so there was no interview non-participation among intervention completers. Interviews were conducted by an occupational therapist who was independent of OPC intervention delivery and had been specifically prepared for the qualitative interview protocol. Analysis retained the Arabic transcripts as the primary analytic source, distinguished participant speech from interviewer prompts, considered prompting context during interpretation, and deliberately retained accounts that challenged more positive patterns. Joint reflexive notes, peer debriefing, discussion of independently coded transcripts, and an audit trail supported scrutiny of developing interpretations. COREQ guided reporting (27).

Several limitations should also shape interpretation. The sample was small, consisted entirely of mothers, and included only families who completed the intervention. Experiences of parents who withdrew before or during treatment, declined participation, or could not meet the digital requirements may differ, creating a likely positive-selection boundary around acceptability and usability. Accordingly, the favorable accounts of usability and acceptability should be interpreted specifically as experiences of intervention completers and should not be generalized to families who were unable to access or complete telehealth. Intervention fidelity was also examined in only three randomly sampled recorded sessions from three different participants at prespecified early and late points. These data provide targeted evidence that the sampled sessions met the prespecified fidelity criterion, but they do not constitute fidelity assessment of every intervention session. The principal investigator was also the intervention coach and led most of the analysis; although the interviewer was independent of OPC delivery, she had prior study-related contact with participants through administration of the COPM, which may have influenced familiarity and the interview interaction. Reflexive documentation, peer discussion, and review of independently coded transcripts were used, but interpretive influence cannot be eliminated. One interview included a participating husband alongside the mother, which may have affected what was discussed even though speaker attribution was considered during analysis. Interviews occurred once after the intervention, so the study cannot establish whether perceived changes or parent problem-solving were sustained. Only one mother elected to review her transcript, and participants did not review the final themes. The study did not include waitlist parents, service providers, or families unable to access videoconferencing. Finally, perceived parent and child changes were self-reported within interviews and should not be interpreted as independent evidence of intervention effectiveness.

## 5 Conclusion

Palestinian mothers experienced telehealth-delivered OPC as a collaborative process that helped them reason through everyday participation challenges, develop strategies that fitted family routines, and take a more active role in action between sessions. Telehealth contributed practical flexibility and reduced reliance on travel, while OPC provided the reflective and occupation-focused process that mothers described as therapeutically useful. These benefits were not cost-free: greater parental ownership required time and energy, digital access was conditional, and perceived progress remained uneven and context-dependent. The findings support further evaluation of OPC telecoaching as a participation-focused complement to pediatric rehabilitation, with implementation designed around caregiver capacity, digital equity, and the realities of the local service environment.

## Data Availability

The interview transcripts are not publicly deposited because they contain detailed contextual information that could increase the risk of participant identification. Requests concerning access to de-identified qualitative material may be directed to the corresponding author and will be considered subject to applicable ethical, institutional, and data-protection requirements. Requests to access the datasets should be directed to mohammad.salahat@aaup.edu.

## References

[1] Graham F Rodger S Ziviani J. Coaching parents to enable children’s participation: an approach for working with parents and their children. Aust Occup Ther J. (2009) 56:16–23. 10.1111/j.1440-1630.2008.00736.x 20854485

[2] Graham F Kennedy-Behr A Ziviani J. Occupational Performance Coaching: A Manual for Practitioners and Researchers. London: Routledge (2020). 10.4324/9780429055805

[3] Graham F Kessler D Nott M Bernie C Kanagasabai P Barthow CA. A scoping review of coaching in occupational therapy: mapping methods, populations and outcomes. Aust Occup Ther J. (2024) 71:1106–30. 10.1111/1440-1630.12991 39315515 PMC11609348

[4] Kemp P Turnbull AP. Coaching with parents in early intervention: an interdisciplinary research synthesis. Infants Young Child. (2014) 27:305–24. 10.1097/IYC.0000000000000018

[5] Akhbari Ziegler S Hadders-Algra M. Coaching approaches in early intervention and paediatric rehabilitation. Dev Med Child Neurol. (2020) 62:569–74. 10.1111/dmcn.14493 32065385 PMC7187136

[6] Tomeny KR McWilliam RA Tomeny TS. Caregiver-implemented intervention for young children with autism spectrum disorder: a systematic review of coaching components. Rev J Autism Dev Disord. (2020) 7:168–81. 10.1007/s40489-019-00186-7

[7] Little LM Pope E Wallisch A Dunn W. Occupation-based coaching by means of telehealth for families of young children with autism spectrum disorder. Am J Occup Ther. (2018) 72:188–95. 10.5014/ajot.2018.024786 29426380

[8] Wallisch A Little L Pope E Dunn W. Parent perspectives of an occupational therapy telehealth intervention. Int J Telerehabil. (2019) 11:15–22. 10.5195/ijt.2019.6274 31341543 PMC6597151

[9] Jamali AR Alizadeh Zarei M Sanjari MA AkbarFahimi M Saneii SH. Randomized controlled trial of occupation performance coaching for families of children with autism spectrum disorder by means of telerehabilitation. Br J Occup Ther. (2022) 85:308–15. 10.1177/03080226211061492 40337660 PMC12033585

[10] Frater T Dunford C Zumaglini S Graham F Green D. Acceptability and feasibility of online occupational performance coaching for parents of children with disabilities in the UK. Br J Occup Ther. (2025) 88:563–73. 10.1177/03080226251340508 40901643 PMC12399807

[11] Pan CY Kuo TY Kuo FL. Meta-analysis of effectiveness of parent-mediated telehealth interventions in children with autism spectrum disorder. Res Autism Spectr Disord. (2023) 107:102209. 10.1016/j.rasd.2023.102209

[12] Alatar W Knott F Loucas T. The effectiveness of telemedicine in coaching parents of autistic children using naturalistic developmental early interventions: a rapid review. Rev J Autism Dev Disord. (2025) 12:273–89. 10.1007/s40489-023-00393-3

[13] Dababnah S Parish SL. “At a moment, you could collapse”: raising children with autism in the West Bank. Child Youth Serv Rev. (2013) 35:1670–8. 10.1016/j.childyouth.2013.07.007

[14] Dababnah S Bulson K. “On the sidelines”: access to autism-related services in the West Bank. J Autism Dev Disord. (2015) 45:4124–34. 10.1007/s10803-015-2538-y 26219419

[15] Wahdan MM Malak MZ Al-Amer R Ayed A Russo S Berte DZ. Effect of incredible years autism spectrum and language delays program on stress and behavioral management skills among parents of children with autism spectrum disorder in Palestine. J Pediatr Nurs. (2023) 72:45–52. 10.1016/j.pedn.2023.03.018 37037104

[16] Palestinian Central Bureau of Statistics. Household Survey on Information and Communications Technology, 2023: Main Findings Report. Ramallah: Palestinian Central Bureau of Statistics (2024). Available online at: https://microdata.pcbs.gov.ps/PCBS-Metadata-en-v5.2/index.php/catalog/733/related-materials (Accessed August 20, 2026).

[17] United Nations Office for the Coordination of Humanitarian Affairs. Movement and Access in the West Bank|September 2024. Jerusalem: United Nations Office for the Coordination of Humanitarian Affairs (2024). Available online at: https://www.ochaopt.org/content/movement-and-access-west-bank-september-2024 (Accessed August 20, 2026).

[18] Shaw JA Connelly DM Zecevic AA. Pragmatism in practice: mixed methods research for physiotherapy. Physiother Theory Pract. (2010) 26:510–8. 10.3109/09593981003660222 20649500

[19] Sandelowski M. Whatever happened to qualitative description? Res Nurs Health. (2000) 23:334–40. 10.1002/1098-240x(200008)23:4<334::aid-nur9>3.0.co;2-g 10940958 10.1002/1098-240x(200008)23:4<334::aid-nur9>3.0.co;2-g

[20] Colorafi KJ Evans B. Qualitative descriptive methods in health science research. HERD. (2016) 9:16–25. 10.1177/1937586715614171 26791375 PMC7586301

[21] Sekhon M Cartwright M Francis JJ. Acceptability of healthcare interventions: an overview of reviews and development of a theoretical framework. BMC Health Serv Res. (2017) 17:88. 10.1186/s12913-017-2031-8 28126032 PMC5267473

[22] Bronfenbrenner U. The Ecology of Human Development. Cambridge, MA: Harvard University Press (1979).

[23] Braun V Clarke V. Can I use TA? Should I use TA? Should I not use TA? Comparing reflexive thematic analysis and other pattern-based qualitative analytic approaches. Couns Psychother Res. (2021) 21:37–47. 10.1002/capr.12360

[24] Braun V Clarke V. Toward good practice in thematic analysis: avoiding common problems and be(com)ing a knowing researcher. Int J Transgend Health. (2023) 24:1–6. 10.1080/26895269.2022.2129597 36713144 PMC9879167

[25] Dunn W Little LM Pope E Wallisch A. Establishing fidelity of occupational performance coaching. OTJR (Thorofare N J). (2018) 38:96–104. 10.1177/1539449217724755 28821218

[26] Rampin R Rampin V. Taguette: open-source qualitative data analysis. J Open Source Softw. (2021) 6:3522. 10.21105/joss.03522

[27] Tong A Sainsbury P Craig J. Consolidated criteria for reporting qualitative research (COREQ): a 32-item checklist for interviews and focus groups. Int J Qual Health Care. (2007) 19:349–57. 10.1093/intqhc/mzm042 17872937

[28] Graham F Rodger S Ziviani J. Mothers’ experiences of engaging in occupational performance coaching. Br J Occup Ther. (2014) 77:189–97. 10.4276/030802214X13968769798791

[29] Graham F Rodger S Ziviani J Jones V. Strategies identified as effective by mothers during occupational performance coaching. Phys Occup Ther Pediatr. (2016) 36:247–59. 10.3109/01942638.2015.1101043 26642938

[30] Lage CR Wright S Monteiro RGDS Aragão L Boshoff K. Collaborative practice with parents in occupational therapy for children: a scoping review. Aust Occup Ther J. (2024) 71:833–50. 10.1111/1440-1630.12974 38923569

[31] Shahbazi M Hojati Abed E Karamali Esmaili S. Family-centered occupational therapy consultation for children under 18 years old: a scoping review. Occup Ther Int. (2026) 2026:1184326. 10.1155/oti/1184326 41522633 PMC12780857

[32] Ashburner J Vickerstaff S Beetge J Copley J. Remote versus face-to-face delivery of early intervention programs for children with autism spectrum disorders: perceptions of rural families and service providers. Res Autism Spectr Disord. (2016) 23:1–14. 10.1016/j.rasd.2015.11.011

[33] Adedipe DT Walton KM. Telehealth parent training for challenging behavior in children with developmental disabilities: a systematic review and meta-analysis. Rev J Autism Dev Disord. (2025). 10.1007/s40489-025-00501-5

[34] Shin Y Park EJ Lee A. Early intervention for children with developmental disabilities and their families via telehealth. Systematic review. J Med Internet Res. (2025) 27:e66442. 10.2196/66442 39819975 PMC11786141

[35] Smith SL Vaquerano J Humphreys BP Aytur SA. Parent satisfaction with a telehealth parent coaching intervention to support family participation. OTJR (Thorofare N J). (2023) 43:531–9. 10.1177/15394492231164942 37122160

[36] Pickard KE Wainer AL Bailey KM Ingersoll BR. A mixed-method evaluation of the feasibility and acceptability of a telehealth-based parent-mediated intervention for children with autism spectrum disorder. Autism. (2016) 20:845–55. 10.1177/1362361315614496 26862084

[37] El-Jardali F Bou-Karroum L Jabbour M Bou-Karroum K Aoun A Salameh S. Digital health in fragile states in the Middle East and North Africa (MENA) region: a scoping review of the literature. PLoS One. (2023) 18:e0285226. 10.1371/journal.pone.0285226 37115778 PMC10146476

[38] Choi D Yu A Kim M Kim EY. Tele-coaching Korean parents for improving occupational performance of toddlers: three case reports. Children. (2023) 10:492. 10.3390/children10030492 36980051 PMC10047845

