# Supplementary material for: Experiences of telehealth-delivered occupational performance coaching among Palestinian mothers of autistic children: a qualitative study
Source: Front Pediatr. 2026 Oct 7;14:1974022. doi: 10.3389/fped.2026.1974022 (PMC13683680; doi:10.3389/fped.2026.1974022)
Supplement: Supplementary file 1 [file Supplementaryfile1.docx]

**Supplementary Material 1**

**Semi-structured interview guide**

*English translation of the original Arabic interview guide*

Reporting note. The interviews were conducted in Arabic. This document provides an English translation of the original Arabic semi-structured interview guide for transparent reporting. The guide preserved the original section order, questions, and examples. Follow-up probes were used flexibly according to participants' responses. The guide indicated an anticipated interview duration of 30–45 minutes; actual interviews lasted approximately 35–60 minutes.

**Introduction**

Thank you for agreeing to speak with me today. This interview aims to understand your experience of the telecoaching program provided to parents of children with autism by Mr. Mohammad Salahat. We would like to learn what worked well, what challenges you experienced, and how the program could be improved. Your responses will remain confidential and will be used to improve services for other families. The interview is expected to take approximately 30–45 minutes.

**Section 1. Research procedures and participation**

I would like to hear about your experience of the telecoaching program in general.

**1. Eligibility and joining the study**

**•** How did you first hear about the telecoaching program?

**•** What helped you decide to participate?

**•** What challenges or difficulties did you experience during registration and consent?

**2. Random allocation and participation**

**•** What did you understand about this process, and did you consider it fair?

**3. Continuation and commitment**

**•** What helped you continue in the program until the end?

**•** What challenges made it difficult to attend sessions or carry out tasks between sessions?

**4. Completing the assessments**

**•** How was your experience completing the online questionnaires and assessments, in terms of ease of understanding, time required, and having a therapist available to assist with questionnaire completion?

**•** How did you feel about identifying goals using the Canadian Occupational Performance Measure (COPM), and how did this help you think about priorities for your child?

**Section 2. Website and digital-platform use**

**1. Accessing and using the website**

**•** How was your experience accessing the Sanad Al-Hayaa website? How was the process of creating an account and registering your child in the program?

**•** Examples: Did you need assistance? Did the instructional videos make the process easier?

**2. Booking sessions**

**•** How was your experience booking sessions using the electronic system? Was it easy or complicated, and why?

**3. Using Zoom**

**•** How comfortable were you using Zoom during the sessions?

**•** What problems did you experience, whether technical problems or internet-connection difficulties?

**4. Using the library and educational materials**

**•** Tell me about your use of the educational materials or the available video library.

**•** Which materials were most helpful?

**5. Suggestions for technological improvement**

**•** What could be improved about using the website or other digital tools?

**Section 3. Program implementation and acceptability**

**1. Conduct of the sessions**

**•** In each session, you and Mr. Mohammad analyzed the goal based on your child’s current and desired performance and the barriers affecting performance. How did you find this approach?

**•** After analyzing the goal, you developed an action plan to apply between sessions. How realistic did you find this plan? Were you able to carry it out? What factors helped or limited your ability to implement it?

**•** During the sessions, how would you describe Mr. Mohammad’s role in the process of analyzing the goal?

**•** How would you describe your own role during the sessions?

**2. Safety and difficulties**

**•** What problems or unexpected situations occurred while taking part in the program?

**3. Overall acceptability**

**•** How would you describe your overall experience of the program?

**•** What did you benefit from most?

**•** What would you like to change or improve?

**Section 4. Perceived impact of the program**

**1. Child participation**

**•** What changes did you notice in your child’s ability to perform everyday activities after the program? Can you share an example of a change that was important to you?

**2. Your confidence and quality of life**

**•** How did the telecoaching intervention affect your confidence in your ability to support your child after participating in the sessions?

**•** What changes did you notice in your family’s way of life, stress levels, or overall family quality of life?

**Section 5. Program format and engagement with content**

**1. Duration and number of sessions**

**•** The program included eight sessions, each lasting about 40 minutes. What did you think about the number and length of the sessions?

**•** Was the session time suitable for you? Would you have preferred shorter, longer, or more sessions?

**Section 6. Comparison with traditional services**

**1. Comparison with centre-based sessions**

**•** If you previously received face-to-face services at a centre, how would you compare that experience with remote coaching sessions?

**•** What differences did you notice in terms of the support provided to you as a mother?

**2. Fit with everyday family life**

**•** Did you feel that remote coaching focused on your daily routines and real-life needs?

**•** How would you compare this with centre-based sessions in terms of linking goals to the child’s everyday life?

**3. Cultural fit and privacy**

**•** How would you describe the remote coaching sessions in terms of their fit with your culture, values, and family privacy?

**•** How did you feel about communicating with the therapist remotely from home rather than attending a centre?

**•** Were there situations in which you felt uncomfortable?

**4. Suggestions for strengthening cultural acceptability**

**•** What could be improved to make this type of program more acceptable in your community?

**Section 7. Practical and logistical aspects**

**1. Time and flexibility**

**•** How did remote coaching help you in terms of time?

**2. Costs**

**•** How did it affect expenses such as transportation, the need for childcare, or taking time away from work?

**3. Health and political challenges**

**•** How did remote delivery help in dealing with difficulties such as closures or political or health-related circumstances?

**4. Effort and pressure**

**•** How would you compare the effort required to participate in this program with services provided at a centre?

**Closing questions**

**•** Would you recommend this program to other parents? What advice would you give to a mother who is newly joining the program?

Thank you very much for your time and openness. Your participation is very important and will contribute to improving services provided to other families in the future.
