# Supplementary material for: Experiences of telehealth-delivered occupational performance coaching among Palestinian mothers of autistic children: a qualitative study
Source: Front Pediatr. 2026 Oct 7;14:1974022. doi: 10.3389/fped.2026.1974022 (PMC13683680; doi:10.3389/fped.2026.1974022)
Supplement: Supplementary file 2 [file Supplementaryfile2.docx]

**Supplementary Material 2. COREQ Checklist**

Note. Section-based locators are used because manuscript pagination may change during production. “Reported” indicates that the item is described in the manuscript; “Not undertaken” indicates that the procedure was not used in the study.

| **Item** | **COREQ topic** | **Guide/reporting response** | **Status** | Section/location in manuscript |
| --- | --- | --- | --- | --- |
| 1 | Interviewer/facilitator | Amira Awda conducted all interviews. | Reported | Methods, 2.4 Research team and reflexivity |
| 2 | Credentials | BSc in Occupational Therapy. | Reported | Methods, 2.4 Research team and reflexivity |
| 3 | Occupation | Occupational therapist. | Reported | Methods, 2.4 Research team and reflexivity |
| 4 | Gender | Female. | Reported | Methods, 2.4 Research team and reflexivity |
| 5 | Experience and training | Substantial experience interviewing parents through university and professional practice; received study-specific training in the qualitative interview protocol from the principal investigator and piloted the interview with one volunteer parent before formal data collection. | Reported | Methods, 2.4 Research team and reflexivity |
| 6 | Relationship established | The interviewer had prior study-related contact with participants through administration of the Canadian Occupational Performance Measure (COPM) in the parent trial but was not involved in delivering the OPC intervention. | Reported | Methods, 2.4 Research team and reflexivity |
| 7 | Participant knowledge of interviewer | Participants knew the interviewer as an occupational therapist involved in study assessment and were informed that she would conduct an interview about their experiences of the program. | Reported | Methods, 2.4 Research team and reflexivity |
| 8 | Interviewer characteristics/reflexivity | The interviewer's prior COPM assessment contact and independence from OPC intervention delivery are reported and considered in relation to data collection. The principal investigator's dual role as intervention provider and primary analyst is also addressed through joint reflexive notes and other reflexive procedures. | Reported | Methods, 2.4 Research team and reflexivity |
| 9 | Methodological orientation and theory | Pragmatic parent-study orientation; qualitative description; reflexive thematic analysis; TFA as a sensitizing framework; OPC/family-centred practice and ecological perspectives used for interpretation. | Reported | Methods, 2.1 Study design, methodological orientation, and theoretical positioning |
| 10 | Sampling | All 12 OPC intervention completers were invited to participate; no separate maximum-variation qualitative sample was constructed. | Reported | Methods, 2.3 Participants and sampling |
| 11 | Method of approach | Participants were informed during the final OPC session; the independent interviewer subsequently contacted each mother, arranged a convenient time, and the mother booked the interview through the Sanad Al-Hayaa platform. Parent-trial recruitment used the Superhero Autism Foundation as a recruitment-outreach partner and social media/autism community pages. | Reported | Methods, 2.3 Participants and sampling |
| 12 | Sample size | 12 mothers who completed the eight-session OPC intervention. | Reported | Methods, 2.2 Parent study and OPC telecoaching intervention; 2.3 Participants and sampling |
| 13 | Non-participation | All 12 OPC completers invited to interview agreed; no qualitative non-participation occurred among intervention completers. | Reported | Methods, 2.3 Participants and sampling |
| 14 | Setting of data collection | Arabic-language interviews conducted remotely via Zoom. | Reported | Methods, 2.5 Interview guide and data collection |
| 15 | Presence of non-participants | One husband/father was present and participated in parts of one interview; he was not an additional participant. Clearly attributable paternal contributions were retained in analysis, joint contributions remained jointly attributed, and ambiguous turns were not reassigned to the mother. | Reported | Methods, 2.3 Participants and sampling; Results, 3 Results |
| 16 | Description of sample | The qualitative sample comprised 12 mothers. Selected maternal and child characteristics are summarized in Table 1; parent-reported autism severity is explicitly distinguished from CARS-2 classifications in the parent trial. | Reported | Methods, 2.3 Participants and sampling; Results, 3 Results; Table 1 |
| 17 | Interview guide | A semi-structured Arabic interview guide informed by TFA is described, including domains and pilot testing with one non-sample parent. The complete English translation is provided as Supplementary Material 1. | Reported | Methods, 2.5 Interview guide and data collection; Supplementary Material 1 |
| 18 | Repeat interviews | None; one interview was rescheduled. | Reported | Methods, 2.5 Interview guide and data collection |
| 19 | Audio/visual recording | Interviews were audio-recorded with permission. | Reported | Methods, 2.5 Interview guide and data collection |
| 20 | Field notes | Reflexive notes were maintained jointly by the principal investigator and Hatem Ahmad Al Adam during transcription and analysis, documenting developing assumptions, interpretive questions, alternative readings, and issues requiring further examination. | Reported | Methods, 2.4 Research team and reflexivity; 2.6 Data analysis |
| 21 | Duration | Approximately 35-60 minutes. | Reported | Methods, 2.5 Interview guide and data collection |
| 22 | Data saturation | Saturation was not used to determine sample size or stop recruitment because all intervention completers were invited. | Reported | Methods, 2.3 Participants and sampling |
| 23 | Transcripts returned | Transcript review was offered to all participants; most declined and one mother reviewed and confirmed her transcript. | Reported | Methods, 2.5 Interview guide and data collection |
| 24 | Number of data coders | The principal investigator coded all interviews. Hatem Ahmad Al Adam independently coded two transcripts and discussed coding and interpretation with the principal investigator for reflexive dialogue and conceptual clarification rather than inter-rater reliability or forced consensus. | Reported | Methods, 2.6 Data analysis |
| 25 | Description of coding tree | The analytic process and development of the final themes and subthemes are described; Hatem Ahmad Al Adam contributed to reviewing emerging codes, final themes, and selected quotations. | Reported | Methods, 2.6 Data analysis; Table 3 |
| 26 | Derivation of themes | TFA provided deductive sensitizing concepts, but themes were not predetermined. Candidate themes were developed iteratively, reviewed against the complete dataset, and refined through analytic discussion, including review of emerging codes and final themes by Hatem Ahmad Al Adam. | Reported | Methods, 2.1 Study design, methodological orientation, and theoretical positioning; 2.6 Data analysis; Table 3 |
| 27 | Software | Taguette supported coding, memo-writing, categorization, and organization of coded segments. | Reported | Methods, 2.6 Data analysis |
| 28 | Participant checking | Participants were not asked to validate the final themes. | Not undertaken | Methods, 2.5 Interview guide and data collection |
| 29 | Quotations presented | Illustrative participant quotations are presented and identified using participant study codes; the final thematic structure is summarized in Table 3. | Reported | Results, 3.1-3.5; Table 3 |
| 30 | Data and findings consistent | Each theme is supported by participant accounts, with interpretive summaries tied to quotations and cross-case variation. | Reported | Results, 3.1-3.5; Table 3 |
| 31 | Clarity of major themes | Five major themes are presented as Results subsections, synthesized in Figure 1, and summarized in Table 3. | Reported | Results, 3.1-3.5; Table 3; Figure 1 |
| 32 | Clarity of minor/diverse cases | Divergent and limiting accounts are reported, including pre-existing confidence, continued value of centre-based care, caregiver workload, conditional digital access, incomplete progress, and variable family-level effects. | Reported | Results, 3.1-3.5 |

Note. Section-based locators are used because manuscript pagination may change during production. “Reported” indicates that the item is described in the manuscript; “Not undertaken” indicates that the procedure was not used in the study.
